# IMGT^®^, the international ImMunoGeneTics information system^®^ 25 years on

**DOI:** 10.1093/nar/gku1056

**Published:** 2014-11-05

**Authors:** Marie-Paule Lefranc, Véronique Giudicelli, Patrice Duroux, Joumana Jabado-Michaloud, Géraldine Folch, Safa Aouinti, Emilie Carillon, Hugo Duvergey, Amélie Houles, Typhaine Paysan-Lafosse, Saida Hadi-Saljoqi, Souphatta Sasorith, Gérard Lefranc, Sofia Kossida

**Affiliations:** IMGT^®^, the international ImMunoGeneTics information system^®^, Université de Montpellier, Laboratoire d'ImmunoGénétique Moléculaire LIGM, UPR CNRS 1142, Institut de Génétique Humaine IGH, 141 rue de la Cardonille, Montpellier, 34396 cedex 5, France

## Abstract

IMGT^®^, the international ImMunoGeneTics information system^®^(http://www.imgt.org) is the global reference in immunogenetics and immunoinformatics. By its creation in 1989 by Marie-Paule Lefranc (Université de Montpellier and CNRS), IMGT^®^ marked the advent of immunoinformatics, which emerged at the interface between immunogenetics and bioinformatics. IMGT^®^ is specialized in the immunoglobulins (IG) or antibodies, T cell receptors (TR), major histocompatibility (MH) and proteins of the IgSF and MhSF superfamilies. IMGT^®^ is built on the IMGT-ONTOLOGY axioms and concepts, which bridged the gap between genes, sequences and 3D structures. The concepts include the IMGT^®^ standardized keywords (identification), IMGT^®^ standardized labels (description), IMGT^®^ standardized nomenclature (classification), IMGT unique numbering and IMGT Colliers de Perles (numerotation). IMGT^®^ comprises 7 databases, 17 online tools and 15 000 pages of web resources, and provides a high-quality and integrated system for analysis of the genomic and expressed IG and TR repertoire of the adaptive immune responses, including NGS high-throughput data. Tools and databases are used in basic, veterinary and medical research, in clinical applications (mutation analysis in leukemia and lymphoma) and in antibody engineering and humanization. The IMGT/mAb-DB interface was developed for therapeutic antibodies and fusion proteins for immunological applications (FPIA). IMGT^®^ is freely available at http://www.imgt.org.

## INTRODUCTION

IMGT^®^, the international ImMunoGeneTics information system^®^ (http://www.imgt.org) (1), was created in 1989 by Marie-Paule Lefranc at Montpellier, France (Université de Montpellier and CNRS). The founding of IMGT^®^ marked the advent of immunoinformatics, a new science, which emerged at the interface between immunogenetics and bioinformatics (2). For the first time, immunoglobulin (IG) or antibody and T cell receptor (TR) variable (V), diversity (D), joining (J) and constant (C) genes were officially recognized as ‘genes’ as well as the conventional genes (3–6). This major breakthrough allowed genes and data of the complex and highly diversified adaptive immune responses to be managed in genomic databases and tools.

IMGT^®^ manages the diversity and complexity of the IG and TR genes and proteins and the polymorphism of the major histocompatibility (MH) proteins of humans and other vertebrates. IMGT^®^ is also specialized in the other proteins of the immunoglobulin superfamily (IgSF) and MH superfamily (MhSF) and related proteins of the immune system (RPI) of vertebrates and invertebrates (1). IMGT^®^ provides a common access to standardized data from genome, proteome, genetics, two-dimensional (2D) and three-dimensional (3D) structures. IMGT^®^ is the acknowledged high-quality integrated knowledge resource in immunogenetics for exploring immune functional genomics.

**Figure 1.**
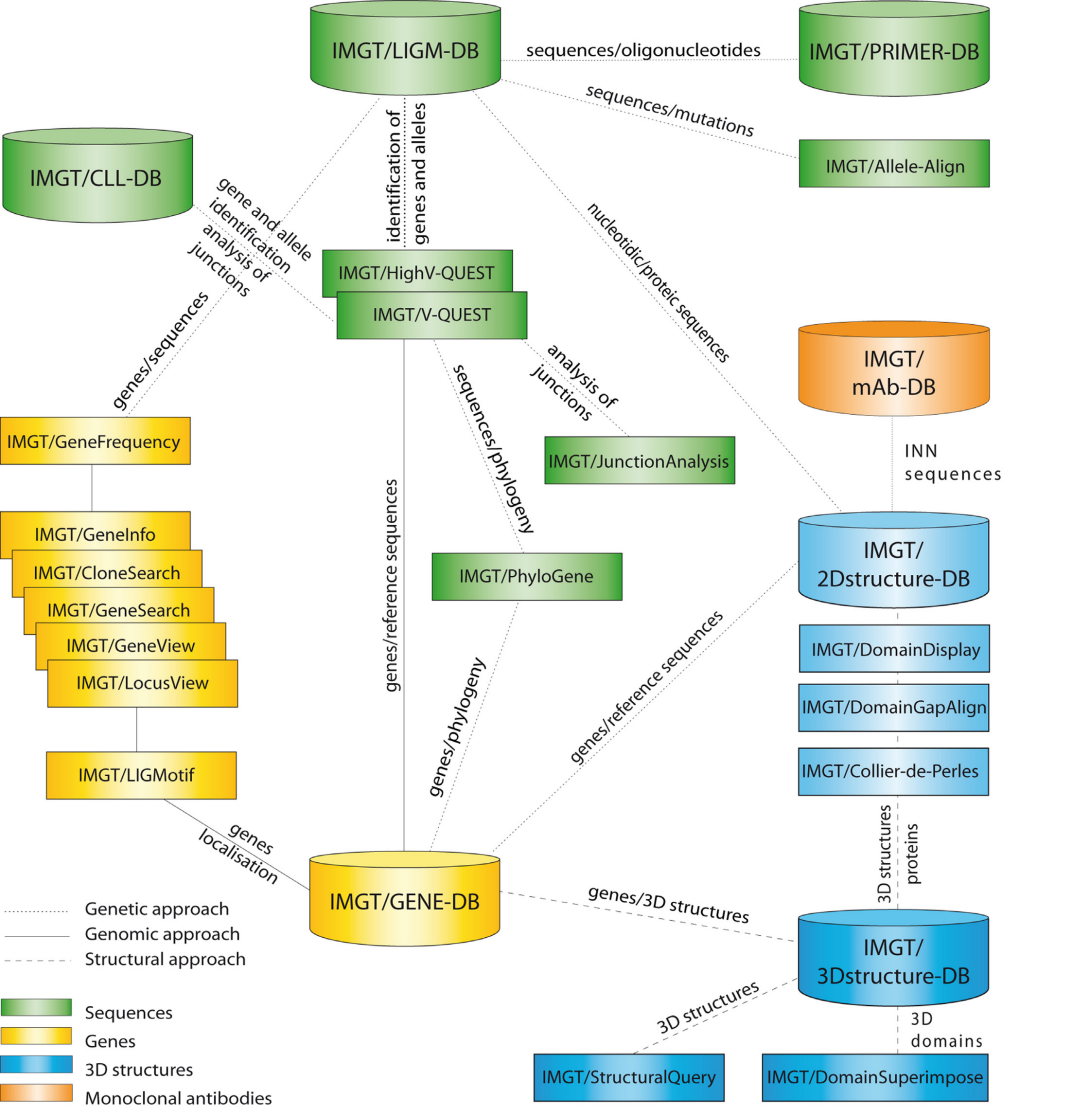
IMGT^®^, the international ImMunoGeneTics information system^®^, http://www.imgt.org. Databases are shown as cylinders and tools as rectangles. The web resources are not shown.

IMGT^®^ comprises 7 databases (7–12), 17 online tools (13–28) and more than 15 000 pages of web resources (Figure 1). Databases include IMGT/LIGM-DB (175 898 entries from 346 species) (7), IMGT/CLL-DB and IMGT/PRIMER-DB for nucleotide sequences and their translation, IMGT/GENE-DB (3431 genes, 5079 alleles) (8) for genes, IMGT/3Dstructure-DB and IMGT/2Dstructure-DB (3682 entries) (9–11) for structures and IMGT/mAb-DB (488 entries) (12) for therapeutic antibodies and fusion proteins for immunological applications (FPIA). The tools are for sequence, gene and structure analysis (13–28) (Figure 1). The web resources include several major sections, for example, IMGT Scientific chart, IMGT Repertoire, IMGT Education > Aide-mémoire (29), The IMGT Medical page, The IMGT Veterinary page, The IMGT Biotechnology page, The IMGT Immunoinformatics page (1).

IMGT^®^ is the global reference in immunogenetics and immunoinformatics (30–36). Its standards have been endorsed by the World Health Organization-International Union of Immunological Societies (WHO-IUIS) Nomenclature Committee since 1995 (first IMGT^®^ online access at the 9th International Congress of Immunology, San Francisco, USA) (37,38) and the WHO International Nonproprietary Names (INN) Programme (39,40) for the description of therapeutic antibodies.

## IMGT-ONTOLOGY

The accuracy and the consistency of the IMGT^®^ data are based on IMGT-ONTOLOGY (41–43), the first, and so far, unique ontology for immunogenetics and immunoinformatics. IMGT-ONTOLOGY manages the immunogenetics knowledge through diverse facets that rely on seven axioms: IDENTIFICATION, DESCRIPTION, CLASSIFICATION, NUMEROTATION, LOCALIZATION, ORIENTATION and OBTENTION (42). The concepts generated from these axioms led to the elaboration of the IMGT^®^ standards that constitute the IMGT Scientific chart: e.g. IMGT® standardized keywords (IDENTIFICATION axiom and concepts of identification) (44), IMGT^®^ standardized labels (DESCRIPTION axiom and concepts of description) (45), IMGT^®^ standardized gene and allele nomenclature (CLASSIFICATION axiom and concepts of classification) (46), IMGT unique numbering (47–52) and its standardized graphical 2D representation or IMGT Colliers de Perles (53–57) (NUMEROTATION axiom and concepts of numerotation).

### IDENTIFICATION: IMGT^®^ standardized keywords

More than 325 IMGT^®^ standardized keywords (189 for sequences and 137 for 3D structures) were precisely defined (44). They represent the controlled vocabulary assigned during the annotation process and allow standardized search criteria for querying the IMGT^®^ databases and for the extraction of sequences and 3D structures. They have been entered in BioPortal at the National Center for Biomedical Ontology (NCBO) in 2010 (http://bioportal.bioontology.org/ontologies/IMGT-ONTOLOGY).

Standardized keywords assigned to a nucleotide sequence are found in the ‘DE’ (definition) and ‘KW’ (keyword) lines of the IMGT/LIGM-DB files (7). They characterize for instance the gene type, the configuration type and the functionality type (44). There are six gene types: variable (V), diversity (D), joining (J), constant (C), conventional-with-leader, conventional-without-leader. Four of them (V, D, J and C) identify the IG and TR genes and are specific to immunogenetics. There are four configuration types: germline (for the V, D and J genes before DNA rearrangement), rearranged (for the V, D and J genes after DNA rearrangement), partially rearranged (for D gene after only one DNA rearrangement) and undefined (for the C gene and for the conventional genes which do not rearrange). The functionality type depends on the gene configuration. The functionality type of genes in germline or undefined configuration is functional (F), ORF (for ‘open reading frame’) or pseudogene (P). The functionality type of genes in rearranged or partially rearranged configuration is either productive (no stop codon in the V-(D)-J region and in-frame junction) or unproductive (stop codon(s) in the V-(D)-J region, and/or out-of-frame junction).

The 20 usual amino acids (AA) have been classified in 11 IMGT physicochemical classes (IMGT^®^ http://www.imgt.org, IMGT Education > Aide-mémoire > Amino acids). The AA changes are identified according to the hydropathy (3 classes), volume (5 classes) and IMGT physicochemical classes (11 classes) (29). For example Q1 > E (+ + −) means that in the AA change (Q > E), the two AA at codon 1 belong to the same hydropathy (+) and volume (+) classes but to different IMGT physicochemical properties (−) classes (29). Four types of AA changes are identified in IMGT^®^: very similar (+ + +), similar (+ + −, + − +), dissimilar (− − +, − + −, + − −) and very dissimilar (− − −).

### DESCRIPTION: IMGT® standardized labels

More than 560 IMGT^®^ standardized labels (277 for sequences and 285 for 3D structures) were precisely defined (45). They are written in capital letters (no plural) to be recognizable without creating new terms. These labels are necessary for a standardized description of the IG, TR and MH sequences and structures in databases and tools (45). Standardized labels assigned to the description of sequences are found in the ‘FT’ (feature) lines of the IMGT/LIGM-DB files (7). Querying these labels represent a big plus compared to the generalist databases (GenBank/European Nucleotide Archive/DNA Data Bank of Japan). Thus, it is possible to query for the ‘CDR3-IMGT’ of the human rearranged productive sequences of IG-Heavy-Gamma (e.g. 1788 CDR3-IMGT obtained, with their sequences at the nucleotide or AA level). There are four core labels for IG and TR, that are V-REGION, D-REGION, J-REGION and C-REGION and which correspond to the coding region of the V, D, J and C genes, respectively. IMGT^®^ structure labels were defined for IG, TR and MH receptors, chains and domains (9–11). A precise and detailed correspondence between structure and sequence labels (2) has contributed to the seamless bridging between sequence and structure data in IMGT^®^ (2) and has strengthened the development of the IMGT domain-centric approach for the V, C and G domains (57).

IMGT^®^ labels were also defined for highly conserved AA at a given position in a domain (2,57). Thus, of the four highly conserved AA between the V and C domains, three have a label: 1st-CYS (cysteine C at position 23), CONSERVED-TRP (tryptophan W at position 41) and 2nd-CYS (C at position 104) (48–50,52,57). In addition, two alternative labels, J-PHE or J-TRP, are characteristics of the IG and TR V-DOMAIN and correspond to the first AA of the canonical F/W-G-X-G motif (where F is phenylalanine, W tryptophan, G glycine and X any AA) encoded by the J-REGION, with F or W being at position 118 (48,49,52,57).

### CLASSIFICATION: IMGT^®^ standardized genes and alleles

The IMGT-ONTOLOGY CLASSIFICATION axiom was the trigger of immunoinformatics’ birth (2). The IMGT^®^concepts of classification allowed, for the first time, to classify the antigen receptor genes (IG and TR) for any locus (e.g. IG heavy (IGH), TR alpha (TRA)), for any gene configuration (germline, undefined or rearranged) and for any species (from fishes to humans) (3–6). In higher vertebrates, there are seven IG and TR major loci (other loci correspond to chromosomal orphon sets, genes of which are orphons, not used in the IG or TR chain synthesis) (3,4). The IG major loci include the IGH, and for the light chains, the IG kappa (IGK) and the IG lambda (IGL) in higher vertebrates (3) and the IG iota (IGI) in fishes (IMGT^®^ http://www.imgt.org, IMGT Repertoire). Since the creation of IMGT^®^ in 1989, at New Haven during the 10th Human Genome Mapping Workshop (HGM10), the standardized classification and nomenclature of the IG and TR of humans and other vertebrate species have been under the responsibility of the IMGT Nomenclature Committee (IMGT-NC).

IMGT^®^ gene and allele names are based on the concepts of classification of ‘Group’, ‘Subgroup’, ‘Gene’ and ‘Allele’ (46). IMGT-ONTOLOGY concepts of classification have been entered in the NCBO BioPortal. New IG and TR genes and alleles are submitted to the IMGT-NC for approval.

The IMGT^®^ IG and TR gene names (2–6) are endorsed by the Human Genome Organisation (HUGO) Nomenclature Committee (HGNC) (58,59) and the WHO-IUIS Nomenclature Subcommittee for IG and TR (37,38). The IMGT^®^ IG and TR gene names are the official international reference and, as such, are entered in IMGT/GENE-DB (8), in Gene (NCBI) (60), in NCBI MapViewer, in Ensembl (61) at the European Bioinformatics Institute and in the Vertebrate Genome Annotation (Vega) Browser (62) at the Wellcome Trust Sanger Institute (UK). HGNC, Gene NCBI, Ensembl and Vega have direct links to IMGT/GENE-DB (8). IMGT^®^ human IG and TR genes were also integrated in IMGT-ONTOLOGY on the NCBO BioPortal and, on the same site, in the HUGO ontology and in the National Cancer Institute Metathesaurus. Since 2007, IMGT^®^ gene and allele names have been used for the description of the therapeutic mAb and FPIA of the WHO-INN programme (39,40).

### NUMEROTATION: IMGT unique numbering and IMGT Collier de Perles

The IMGT-ONTOLOGY NUMEROTATION axiom is acknowledged as the ‘IMGT^®^ Rosetta stone’ that has bridged the biological and computational spheres in bioinformatics (31). The IMGT^®^ concepts of numerotation comprise the IMGT unique numbering (47–52) and its graphical 2D representation the IMGT Collier de Perles (53–57). Developed for and by the ‘domain’, these concepts integrate sequences, structures and interactions into a standardized domain-centric knowledge for functional genomics. The IMGT unique numbering has been defined for the variable V domain (V-DOMAIN of the IG and TR, and V-LIKE-DOMAIN of IgSF other than IG and TR) (47–49), the constant C domain (C-DOMAIN of the IG and TR, and C-LIKE-DOMAIN of IgSF other than IG and TR) (50) and the groove G domain (G-DOMAIN of the MH, and G-LIKE-DOMAIN of MhSF other than MH) (51). Thus, the IMGT unique numbering and IMGT Collier de Perles provide a definitive and universal system across species including invertebrates, for the sequences and structures of the V, C and G domains of IG, TR and MH, and more generally of the IgSF and MhSF superfamilies (57).

## INTERACTION BETWEEN IMGT^®^ DATABASES AND TOOLS

IMGT^®^ comprises 7 databases and 17 online tools for sequences, genes and structures which have been described in details, previously (7–28). Links to documentation, releases and statistics are available from the IMGT^®^ Home page, http://www.imgt.org. Here, we will focus mainly on examples demonstrating the strong interactions which exist between IMGT^®^ databases and tools, based on the IMGT^®^ rules and standards, generated from the IMGT-ONTOLOGY axioms and concepts and described in the IMGT Scientific chart. First, we will describe briefly the IMGT^®^ reference directory databases that support the most popular tools for sequence analysis, then we will describe how the IMGT/3Dstructure-DB and IMGT/2Dstructure-DB databases are intimately associated with tool functionalities/results. These databases that bridge the gap between AA sequences and 3D structures can also be accessed by querying the IMGT/mAb-DB interface.

### IMGT^®^ reference directory databases

#### IMGT/V-QUEST reference directory

IMGT/V-QUEST (13–18) and its high-throughput version, IMGT/HighV-QUEST (18,23,24) analyse nucleotide sequences of the IG and TR variable domains. These tools run against the IMGT/V-QUEST reference directory database (Table 1) that includes several sets. These sets comprise IMGT reference sequences from all functional (F) genes and alleles, all ORF and all in-frame pseudogenes (P) alleles from IMGT/GENE-DB (8). By definition, the IMGT reference directory sets contain one sequence for each allele. By default, the user sequences are compared with all genes and alleles. However, the IMGT/V-QUEST and IMGT/HighV-QUEST option ‘With allele *01 only’ can be useful if the user sequences need to be compared with different genes or if the user sequences that use the same gene need to be aligned together (independently of the allelic polymorphism) (13–18).

**Table 1.** IMGT^®^ reference directory databases for IMGT^®^ tools for analysis of V, C or G domains (nucleotide and AA sequences)

IMGT^®^ reference directory databases	IMGT^®^ tools	Entry types and examples of applications	Results for V, C or G domains^a^
IMGT/V-QUEST reference directory	IMGT/V-QUEST (13–18)	User nucleotide sequences of V-DOMAIN (1 to 50 sequences per analysis, and 1 to 10 sequences with the option ‘Search for insertions and deletions’) (17)	1. Introduction of IMGT gaps
		***Applications:*** somatic mutations in chronic lymphocytic leukemia (CLL) prognostic.	2. Identification of the closest germline V, D and J genes and alleles
			3. IMGT/JunctionAnalysis results (19,20)
			4. Translation in different formats
			5. Description of mutations and AA changes
			6. Identification of indels and their correction (17) (option).
			7. IMGT/Automat annotation (21,22)
			8. IMGT Colliers de Perles (27).
	IMGT/HighV-QUEST (18,23,24)	User NGS nucleotide sequences of V-DOMAIN (up to 500,000 sequences per run)^b^ (23,24)	1. Introduction of IMGT gaps
		***Applications:*** IG and TR immune repertoires and clonotypes in NGS.	2. Identification of indels and their correction (18) (by default)
			3. Identification of the closest germline V, D and J genes and alleles
			4. Translation in different formats
			5. IMGT/JunctionAnalysis results (19,20)
			6. Description of mutations and AA changes
			7. IMGT/Automat annotation (21,22)
			8. Statistical analysis (24)
			9. Characterization of the IMGT clonotypes (AA), clonal diversity and expression (24).
IMGT/DomainSeq reference directory	IMGT/DomainGapAlign (10,25,26)	User AA sequences of V, C and G domains (one to several sequences of same domain type) (25,26)	1. Introduction of IMGT gaps
		***Applications:*** IMGT antibody engineering and humanization for V and C.	2. Identification of the closest genes and alleles (germline V and J for V-DOMAIN)
			3. Delimitation of the domains
			4. Description of AA changes
			5. IMGT Colliers de Perles (53–57) with highlighted AA changes (pink circles online).

^a^V: V domain (includes V-DOMAIN of IG and TR and V-LIKE-DOMAIN of IgSF other than IG and TR) (48,49).

C: C domain (includes C-DOMAIN of IG and TR and C-LIKE-DOMAIN of IgSF other than IG and TR) (50).

G: G domain (includes G-DOMAIN of MH and G-LIKE-DOMAIN of MhSF other than MH) (51).

^b^in September 2014, more than 4 billions of sequences analyzed by IMGT/HighV-QUEST, by 943 users from 40 countries (45% users from USA, 36% from EU, 19% from the remaining world).

The IMGT/V-QUEST reference directories have been set up for species which have been extensively studied, such as human and mouse. This also holds for the other species or taxons with incomplete IMGT reference directory sets. In those cases, results should be interpreted considering the status of the IMGT reference directory (information on the updates on the IMGT^®^ web site). Links to the IMGT/V-QUEST reference directory sets are available from the IMGT/V-QUEST Welcome page (13–18).

The analysis of the junctions of the rearranged V-J and V-D-J sequences of the IG and TR variable domains (3,4) is performed by the IMGT/JunctionAnalysis tool (19,20) which is integrated in IMGT/V-QUEST and IMGT/HighV-QUEST. This tool provides a detailed analysis by delimiting very precisely the different regions that participate to the junction. To answer this higher-resolution analysis, additional labels (3′V-REGION, 5′J-REGION) and corresponding reference directory sets had to be created.

#### IMGT/DomainSeq reference directory

IMGT/Domain GapAlign (10,25,26) analyses AA sequences of the V, C and G domains. This tool run against the IMGT/DomainSeq reference directory database (Table 1) that include sets for the V, C and G domains. These sets comprise sequences from the IMGT Repertoire (1) and from IMGT/GENE-DB (8). Owing to the particularities of the IG and TR V-DOMAIN synthesis (3,4) there is no V-DOMAIN in the IMGT/DomainSeq reference directory. Instead, the directory comprises the translation of the IG and TR germline V and J genes (V-REGION and J-REGION, respectively). The IMGT/DomainSeq reference directory provides the IMGT^®^ ‘gene’ and ‘allele’ names. Data are comprehensive for human and mouse IG and TR, whereas for other species and other IgSF and MhSF they are added progressively. The IMGT/DomainSeq reference directory comprises domain sequences of functional (F), ORF and in-frame pseudogene (P) genes. As IMGT^®^ alleles are characterized at the nucleotide level, identical sequences at the AA level may therefore correspond to different alleles, in the IMGT/DomainSeq reference directory. The sequences of the IMGT/DomainSeq reference directory sets can be displayed by querying IMGT/DomainDisplay (http://www.imgt.org).

### IMGT structure databases

#### IMGT/3Dstructure-DB

IMGT/3Dstructure-DB (9–11), the IMGT^®^ structure database, provides IMGT^®^ annotation and contact analysis of IG, TR, MH, IgSF and MhSF 3D structures, and paratope/epitope description of IG/antigen (32,33,35,63–66) and TR/pMH (67,68) complexes (Table 2). There is one ‘IMGT/3Dstructure-DB card’ per IMGT/3Dstructure-DB entry and this card provides access to all data related to that entry. The ‘Protein Data Bank (PDB) code’ (4 letters and/or numbers, e.g. 1n0x) is used as ‘IMGT entry ID’ for the 3D structures obtained from the Research Collaboratory for Structural Bioinformatics PDB (69)**.** The IMGT/3Dstructure-DB card provides eight search/display options: ‘Chain details’, ‘Contact analysis’, ‘Paratope and epitope’, ‘3D visualization Jmol or QuickPDB’, ‘Renumbered IMGT files’, ‘IMGT numbering comparison’, ‘References and links’, ‘Printable card’ (9–11).

**Table 2.** IMGT^®^ structure databases and associated tool functionalities/results

IMGT^®^ structure databases	Content and examples of applications	Tool functionalities/results (including those for V, C of G domains^a^)
IMGT/3Dstructure-DB (9–11)	3128 structure entries of which 2053 IG (including 1315 IG/Ag complexes), 240 TR and 712 MH (including 143 TR/pMH complexes: 108 TR/pMH1 + 35 TR/pMH2) (September 2014).	1. Identification of the closest genes and alleles (germline V and J genes and alleles for V-DOMAIN)
	***Applications:*** identification of the paratope and epitope in IG/AG and TR/pMH complexes and pMH contacts.	2. IMGT/DomainGapAlign results (10,25,26)
		3. IMGT Collier de Perles (53–57) (on two layers with hydrogen bonds for V and C domains or with pMH contact sites for G-DOMAIN)
		4. Contact analysis between a pair of domains or between a domain and a ligand
		5. Renumbered IMGT files
		6. IMGT numbering comparison
IMGT/2Dstructure-DB (11)*	561 AA sequence entries (225 INN + 336 Kabat) of which 548 IG (212 INN + 336 Kabat) (September 2014)*	1. Identification of the closest genes and alleles (germline V and J genes and alleles for V-DOMAIN)
	***Applications:*** from gene to structures in the absence of 3D.	2. IMGT/DomainGapAlign results (10,25,26)
		3. IMGT Collier de Perles (53–57)
		4. Renumbered IMGT files

An asterisk (*) indicates that parts of the protocol dealing with 3D structures (hydrogen bonds in IMGT Colliers de Perles on two layers, Contact analysis) are not relevant, otherwise all other queries and results are similar to IMGT/3Dstructure-DB.

^a^V: V domain (includes V-DOMAIN of IG and TR and V-LIKE-DOMAIN of IgSF other than IG and TR) (48,49).

C: C domain (includes C-DOMAIN of IG and TR and C-LIKE-DOMAIN of IgSF other than IG and TR) (50).

G: G domain (includes G-DOMAIN of MH and G-LIKE-DOMAIN of MhSF other than MH) (51).

The ‘Chain details’ section comprises information first on the chain itself, then per domain (9–11). Chain and domain annotation includes the IMGT gene and allele names (CLASSIFICATION), region and domain delimitations (DESCRIPTION) and domain AA positions according to the IMGT unique numbering (NUMEROTATION) (47–52). The closest IMGT^®^ genes and alleles (found expressed in each domain of a chain) are identified with the integrated IMGT/DomainGapAlign (10,25,26), which aligns the AA sequences of the 3D structures with the IMGT/DomainSeq reference directory.

‘Contact analysis’ gives access to a table with the different ‘Domain pair contacts’ of the 3D structure (this table is also accessed from ‘Chain details’ by clicking on ‘Domain contact (overview)’). ‘Domain pair contacts’ refer to contacts between a pair of domains or between a domain and a ligand. Clicking on ‘DomPair’ gives access to the contacts between AA for a given ‘Domain pair contacts’. For IG/Ag (32,33,35,63–66) and TR/pMH (67,68) complexes, the paratope and epitope are displayed in Contact analysis, but for each V domain, separately.

‘Renumbered IMGT file’ allows to view (or download) an IMGT coordinate file renumbered according to the IMGT unique numbering, and with added IMGT specific information on chains and domains (added in the ‘REMARK 410′ lines (blue online), and identical to the ‘Chain details’ annotation). ‘IMGT numbering comparison’ provides, per domain, the IMGT DOMAIN numbering by comparison with the PDB numbering, and the residue (3-letter and 1-letter names), which allows standardized IMGT representations using generic tools.

#### IMGT/2Dstructure-DB

IMGT/2Dstructure-DB was created as an extension of IMGT/3Dstructure-DB (9–11) to describe and analyse AA sequences of chains and domains for which no 3D structures were available (Table 2). IMGT/2Dstructure-DB uses the IMGT/3Dstructure-DB informatics frame and interface which allow one to analyse, manage and query IG, TR and MH, as well as other IgSF and MhSF and engineered proteins (FPIA, composite proteins for clinical applications (CPCA)), as polymeric receptors made of several chains, in contrast to the IMGT/LIGM-DB sequence database that analyses and manages sequences individually (7). The AA sequences are analysed with the IMGT^®^ criteria of standardized identification (44), description (45), nomenclature (46) and numerotation (47–52).

The current IMGT/2Dstructure-DB entries include AA sequences of antibodies from Kabat (70) (those for which there were no available nucleotide sequences), and AA sequences of mAb and FPIA from the WHO-INN programme (12,39,40). Queries can be made on an individual entry, using the Entry ID or the Molecule name. The same query interface is used for IMGT/2Dstructure-DB and IMGT/3Dstructure-DB. Thus a ‘trastuzumab’ query in ‘Molecule name’ allows to retrieve three results: two INN (‘trastuzumab’ and ‘trastuzumab emtansine’) from IMGT/2Dstructure-DB, and one 3D structure (‘1nz8′) from IMGT/3Dstructure-DB.

The IMGT/2Dstructure-DB cards provide standardized IMGT information on chains and domains and IMGT Colliers de Perles on one or two layers, identical to that provided for the sequence analysis in IMGT/3Dstructure-DB, however, the information on experimental structural data (hydrogen bonds in IMGT Collier de Perles on two layers, Contact analysis) is only available in the corresponding IMGT/3Dstructure-DB cards, if the antibodies have been crystallized.

#### IMGT/mAb-DB

**Figure 2.**
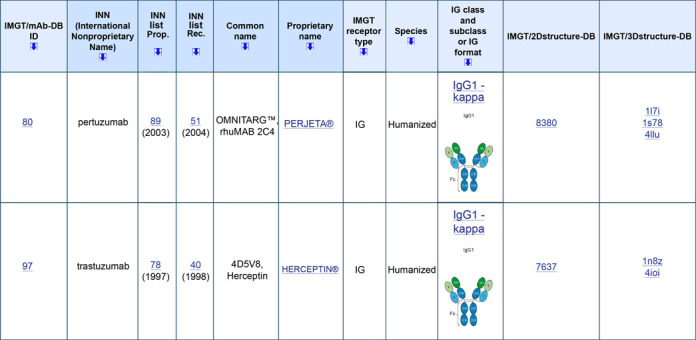
IMGT/mAb-DB, http://www.imgt.org. The part of the results for two entries is shown with the links to IMGT/2Dstructure-DB and IMGT/3Dstructure-DB, respectively.

A new database and interface, IMGT/mAb-DB (12), http://www.imgt.org, has been developed to provide an easy access to therapeutic antibody AA sequences (links to IMGT/2Dstructure-DB) and structures (links to IMGT/3Dstructure-DB, if 3D structures are available) (Figure 2). IMGT/mAb-DB data include monoclonal antibodies (mAb, INN suffix –mab) (a –mab is defined by the presence of at least an IG variable domain) and fusion proteins for immune applications (FPIA, INN suffix –cept) (a –cept is defined by a receptor fused to a Fc) from the WHO-INN programme (39,40). This database also includes a few CPCA (e.g. protein or peptide fused to a Fc for only increasing their half-life, identified by the INN prefix ef–) and some RPI used, unmodified, for clinical applications.

## FUTURE DIRECTIONS

IMGT-ONTOLOGY and the IMGT^®^ information system, which are at the origin of immunoinformatics, have provided the concepts, the knowledge environment and the informatics frame for a standardized and integrated analysis of IG, TR and MH, extended to other IgSF and MhSF, from gene to structure and function (2). IG and TR repertoire and clonality analysis, NGS repertoire in normal immune responses (vaccination, cancers, infections) and in abnormal responses (autoimmune diseases), clonotype specificity, antibody humanization, IG and TR engineering for immunotherapy, IG allotypes and immunogenicity, paratope/epitope characterization and specificity represent major current fields of immunoinformatics at the forefront of basic, clinical and pharmaceutical research owing to major methodological advances and medical implications.

The IMGT^®^ databases and tools, and implicitly IMGT^®^ reference directories, are widely used in clinical applications. Thus, IMGT/V-QUEST is frequently used by clinicians for the analysis of IG somatic hypermutations in leukemia, lymphoma and myeloma, and more particularly in chronic lymphocytic leukemia (CLL) (16,71) in which the percentage of mutations of the rearranged IGHV gene in the VH of the leukemic clone has a prognostic value for the patients. For this evaluation, IMGT/V-QUEST is the standard recommended by the European Research Initiative on CLL for comparative analysis between laboratories (71). The sequences of the V-(D)-J junctions determined by IMGT/JunctionAnalysis (19,20) are also used in the characterization of stereotypic patterns in CLL and for the synthesis of probes specific of the junction for the detection and follow-up of minimal residual diseases (MRD) in leukemias and lymphomas. A new era is opening in hemato-oncology with the use of NGS for analysis of the clonality and MRD identification, making IMGT^®^ standards use more needed as ever. More generally, the IMGT/HighV-QUEST web portal is a paradigm for identification of IMGT clonotype diversity and expression in NGS immune repertoire analysis of the adaptive immune response in infectious diseases, in vaccination and for next generation repertoire immunoprofiling (24). The IMGT^®^ reference directory databases behind these tools are key to provide standardized results.

The therapeutic monoclonal antibody engineering field represents the most promising potential in medicine (64). A standardized analysis of IG genomic and expressed sequences, structures and interactions is crucial for a better molecular understanding and comparison of the mAb specificity, affinity, half-life, Fc effector properties and potential immunogenicity. IMGT-ONTOLOGY concepts have become a necessity for IG loci description of newly sequenced genomes, antibody structure/function characterization, allotypes in relation with molecular and structural analysis (72–74), antibody engineering (single chain Fragment variable (scFv), phage displays, combinatorial libraries) and antibody humanization (chimeric, humanized and human antibodies) (32,33,35,63–66). IMGT^®^ standardization allows repertoire analysis and antibody humanization studies to move to novel high-throughput methodologies with the same high-quality criteria. The CDR-IMGT lengths are now required for mAb INN applications and are included in the WHO-INN definitions (40), bringing a new level of standardized information in the comparative analysis of therapeutic antibodies.

## CITING IMGT

Users are requested to cite this article and quote the IMGT Home page URL, http://www.imgt.org.
